# Supplementary figures and images for: A theoretical morphological model for quantitative description of the three-dimensional floral morphology in water lily (Nymphaea)
Source: PLoS One. 2020 Oct 12;15(10):e0239781. doi: 10.1371/journal.pone.0239781 (PMC7549838; doi:10.1371/journal.pone.0239781)

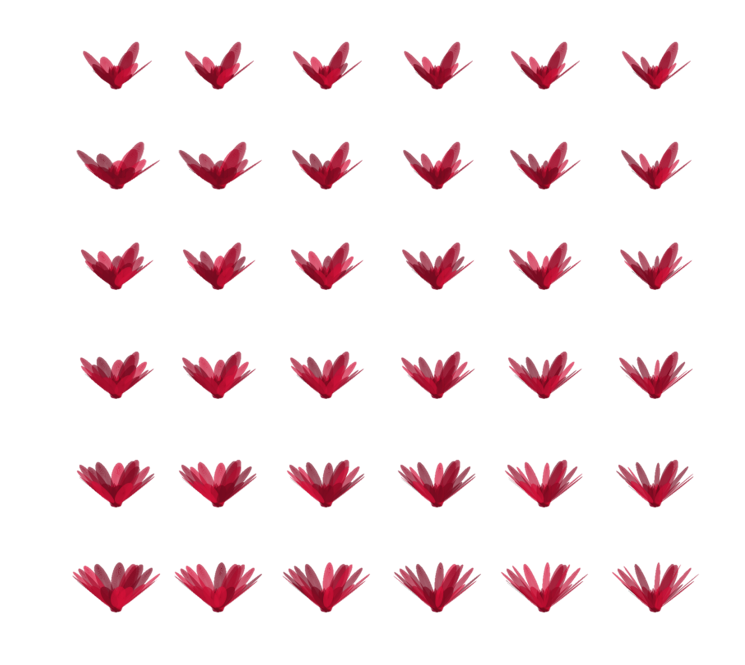

Supplement: S1 Striking image — (TIF) [file pone.0239781.s001.tif]
